# The value of biofilm testing to guide antimicrobial stewardship in chronic respiratory diseases

**DOI:** 10.3389/fcimb.2023.1142274

**Published:** 2023-05-02

**Authors:** Laia Fernández-Barat, Nil Vázquez Burgos, Victoria Alcaraz, Leticia Bueno-Freire, Ruben López-Aladid, Roberto Cabrera, Albert Gabarrús, Andrea Palomeque, Patricia Oscanoa, Adrian Ceccato, Ana Motos, Rosanel Amaro, Thierry Bernardi, Christian Provot, Alba Soler-Comas, Laura Muñoz, Jordi Vila, Antoni Torres

**Affiliations:** ^1^Cellex Laboratory, CibeRes (CB06/06/0028)-Instituto de Investigaciones Biomédicas August Pi i Sunyer (IDIBAPS), School of Medicine, University of Barcelona, Barcelona, Spain; ^2^Pneumology Service, Respiratory Institute, Hospital Clinic, Barcelona, Spain; ^3^BioFilm Pharma SAS, Lyon, France; ^4^BioFilm Control SAS, Saint Beauzire, France; ^5^Microbiology Service, Hospital Clinic, Barcelona, Spain

**Keywords:** *Pseudomonas aeruginosa*, antimicrobial agents, antimicrobial resistances, biofilm diagnose, biofilm

## Abstract

**Introduction:**

Biofilm production is an important yet currently overlooked aspect of diagnostic microbiology that has implications for antimicrobial stewardship. In this study, we aimed to validate and identify additional applications of the BioFilm Ring Test® (BRT) for Pseudomonas aeruginosa (PA) isolates from patients with bronchiectasis (BE).

**Materials and methods:**

Sputa were collected from BE patients who had at least one PA positive culture in the previous year. We processed the sputa to isolate both mucoid and non-mucoid PA, and determined their susceptibility pattern, mucA gene status, and presence of ciprofloxacin mutations in QRDR genes. The Biofilm production index (BPI) was obtained at 5 and 24 hours. Biofilms were imaged using Gram staining.

**Results:**

We collected 69 PA isolates, including 33 mucoid and 36 non-mucoid. A BPI value below 14.75 at 5 hours predicted the mucoid PA phenotype with 64% sensitivity and 72% specificity.

**Conclusion:**

Overall, our findings suggest that the fitness-cost associated with the mucoid phenotype or ciprofloxacin resistance is shown through a time-dependent BPI profile. The BRT has the potential to reveal biofilm features with clinical implications.

## 1 Introduction

Non-cystic fibrosis (non-CF) bronchiectasis (BE) is a chronic structural and inflammatory respiratory disease characterized by irreversible destruction and dilatation of the bronchi that result in recurrent infections and exacerbations. Physiopathologically, the tissular destruction involves a vicious circle of impaired mucociliary clearance, bronchial infection and chronic inflammation (Cohen and Sahn, 1999; Barker, 2002; King et al., 2006; Hill et al., 2017; Polverino et al., 2017; Flume et al., 2018).. *Haemophilus influenzae* and *Pseudomonas aeruginosa* (PA) are the most frequently isolated microorganisms in non-CF BE exacerbations (Chandrasekaran et al., 2018). Furthermore, PA is an independent factor contributing to a threefold increase in the risk of death. It is also associated with a higher number of exacerbations and hospitalizations, and higher symptomatology perceived by the patient (Finch et al., 2015). Given the relationship between PA and poor clinical outcomes in patients with BE, its early detection and appropriate management are essential (Fernández-Barat et al., 2021).

Early PA colonization is frequently associated with the isolation of the non-mucoid phenotype. However, unless eradicated, the non-mucoid strain can shift to a mucoid PA phenotype through genetic changes such as mutations in mucA gene (Fernández-Barat et al., 2017; Crisafulli et al., 2018; Dhand, 2018). These mutations are considered to play an important role in the genetic adaptive evolution of PA. It has been demonstrated that mutator populations are amplified in the lung by presenting adaptive mutations (Ciofu et al., 2017). Loss-of-function mutations in mucA cause an overproduction of alginate exopolysaccharide, which is characteristic of the mucoid phenotype. The mucoid phenotype has been shown to be a hallmark of chronic infections, higher viscoelasticity of sputum (Alcaraz-Serrano et al., 2019) and biofilm maturation, which impair both the effect of antibiotics and host immune functions (Malhotra et al., 2018). In addition, the metabolic rate of PA within biofilm aggregates is lower than its planktonic counterparts, driving bacterial cells to a dormant state in which some of them become “persister cells” that do not divide and are highly tolerant to antimicrobials (Lewis, 2007; Yang et al., 2008).

It is important to highlight that the Minimum Inhibitory Concentration (MIC) routinely reported by the microbiology laboratories does not reflect the Minimum Biofilm Inhibitory Concentration (MBIC) since the MBIC is often several times greater than the MIC of a planktonic PA strain (Ciofu et al., 2017). Thus, the diagnostic value of biofilm formation is currently underestimated hindering the management of patients with chronic respiratory diseases (Wang et al., 2012; Thieme et al., 2019).

Multiple methods are available to measure bacterial biofilm production, although none of them are currently applied in the routine standard of care (Peeters et al., 2008; Pantanella et al., 2013). The microtiter plate method is extensively used to quantify the *in vitro* biofilm capability of bacteria, but it is limited by the inability to confidently extrapolate those results to *in vivo* scenarios (Fernández-Barat et al., 2018). BioFilm Ring Test^®^ (BRT) is a novel technology developed to determine biofilm formation production by the measurement of the adhesion between bacteria. It has shown increased sensitivity and specificity compared to the traditional crystal violet test. The BRT does not require staining, is easy to handle and the results can be obtained in a few hours, being more suitable for clinical practice than previous techniques (Chavant et al., 2007; Sulaeman et al., 2010; Olivares et al., 2016). The BRT has been recently shown to have a potential application in the selection of antimicrobials in CF (Olivares et al., 2016). However, additional applications of the BRT, such as its correlation with microbiological and clinical features have not been previously reported (Di Domenico et al., 2016). We aimed to validate the BRT assay using a significant number of PA clinical isolates from Non-CF BE patients. We also aimed to investigate additional applications of the BRT by comparing the biofilm production index (BPI) between mucoid and non-mucoid PA isolates, susceptible and resistant PA, presence and absence of biofilm pattern by Gram and intermittent and chronic infection status, in addition to assess the sensitivity and specificity for significant associations. Finally, we correlated the BPI with the mutations in mucA gene.

## 2 Material and methods

### 2.1 Definitions

#### 2.1.1 Bronchiectasis

A diagnosis of BE of any cause in the absence of chronic obstructive pulmonary disease (COPD) using high-resolution computed tomography (HRCT) of the chest.

#### 2.1.2 Bronchiectasis with chronic obstructive pulmonary disease

Diagnosis of BE as mentioned above and a diagnosis of COPD (persistent respiratory symptoms and airflow limitation with a history of smoking, according to the GOLD criteria (Vogelmeier et al., 2017).

#### 2.1.3 Chronic infection

≥2 PA isolates in respiratory specimens ≥3 months apart in 1 year (Polverino et al., 2017).

#### 2.1.4 Intermittent infection

PA isolates in respiratory specimens not accomplishing the chronic infection definition (Fernández-Barat et al., 2021).

#### 2.1.5 Exacerbation

Deterioration in three or more key symptoms: cough, sputum volume and/or consistency, sputum purulence, dyspnea and/or exercise tolerance, fatigue or malaise, and hemoptysis, in accordance with European guidelines (Hill et al., 2017).

#### 2.1.6 Slow growing PA

PA with an increased BPI in 24 h of incubation when compared to 5 h of incubation.

### 2.2 Patients and strains

The studies involving human participants were reviewed and approved by the Internal Review Board of the Hospital Clinic of Barcelona (registry number HCB/0236). Written informed consent was obtained from all patients. The study was carried out in compliance with the Declaration of Helsinki (current version: Fortaleza, Brazil, October 2013) and with the requirements of the 2007 Spanish Biomedical Research Act.

All patients (≥18 years) had BE (confirmed by a CT scan) with or without COPD and had at least one recent positive sputum culture for PA prior to study recruitment. Valid sputa were immersed in 1:1 Dithiothreitol (DTT) solution and sonicated for 5 min at 40KHz in an ultrasonic cleaning equipment (Branson 3510 E-MT; Bransonic, Danbury, CT, USA), before being serially diluted in 0.9% saline solution and cultured both in MacConkey agar and Blood agar (BD). PA strains were isolated and identified by MALDI-TOF. They were classified as mucoid or non-mucoid phenotypes depending on the colony morphology. An extension of each fresh sample was obtained for the Gram staining.

### 2.3 Imaging PA biofilms by Gram staining

The quality of the sample was evaluated by Gram staining in the area of maximal purulence, according to the criteria of Murray and Washington (1975). To image the Gram staining, an Olympus BX41TF microscope (Olympus, Tokyo, Japan) with a 100X oil immersion lens was used. Gram-negative bacilli susceptible of PA (PA was confirmed in culture from the same sputum sample) were classified into 3 groups: planktonic PA (Gram-negative rods present without aggregates), PA biofilm (Gram-negative rods present in aggregates) and PA alginate (Gram-negative rods in aggregates embedded in alginate).

### 2.4 Susceptibility testing

Strains were classified as resistant, intermediate or susceptible to amikacin, tobramycin, imipenem, meropenem, ceftazidime, ciprofloxacin, piperacillin-tazobactam, aztreonam, and colistin according to the EUCAST (2017) breakpoints using the disk diffusion method (BD) following the EUCAST protocol. ATCC 27853 was used as quality control. Strains were then categorized as non-multidrug resistant (non-MDR) or resistant strains (including multidrug resistant (MDR) and extensive drug resistant (XDR) strains) according to current definitions (Magiorakos et al., 2012).

### 2.5 Biofilm ring test

The test was performed using the reagents in the Biofilm Ring Test kit (KIT01) (Biofilm Control, Saint Beauzire, France), following Chavant T. et al. protocol (Chavant et al., 2007). Bead aggregation was analyzed by the BFC Elements 3.0 software (Biofilm reader, Biofilm Control, Saint Beauzire, France). In order to minimize variability on the BPI, 8 intra-assay replicates and inter-assay triplicates were performed. PA were then classified into weak BPI<5, moderate BPI≥5 but <10, strong BPI≥10 but <15 and very strong BPI≥15 biofilm producers based on their BPI. In a subset of 20 mucoid and 9 non-mucoid PA suspected of slow biofilm production at 5h, an extended experiment was performed, using the aforementioned protocol but with a 24h incubation instead of the 5h of the standard protocol in order to elucidate the role of the PA slow growth in the BPI results. We classified the strains showing an increase in BPI from 5 to 24 hours as slow-growing *Pseudomonas aeruginosa* strains. To estimate their growth rate, we used the percentage of the total BPI at 5 hours. We then compared the growth rates between mucoid and non-mucoid strains that exhibited slow growth.

### 2.6 Mutations in mucA and QRDR genes

The mucA gene of all PA and for quinolone resistant QRDR genes (gyrA, gyrB, parC and parE) for 43 PA isolates included in this study were amplified by PCR. Primers used for amplification and sequencing are reported in **Table 1** or previously published (Cabrera et al., 2022). PCR products were sequenced by Sanger methods (Genewiz, Germany), and were analyzed by alignment with the corresponding template sequence of PAO1 mucA at GenBank (Ciofu et al., 2010). PCR was performed in a Veriti PCR Thermal Cycler (Applied Biosystems, France) for 2 min denaturation at 94°C followed by 30 cycles of 1 min at 94°C, 1 min at 60°C and 1 min at 72°C, with a final extension of 7 min at 72°C.

**Table 1:** Amplification and sequencing primers for mucA.

*mucA1*	F (5’ 3’)	CTCTGCAGCCTTTGTTGCGAGAAG
*mucA1*	R (5’ 3’)	CTGCCAAGCAAAAGCAACAGGGAGG
*mucA2*	F (5’ 3’)	GTGCGTCTGTACAACCAGAACGACG
*mucA2*	R (5’ 3’)	GTCGTTCTGGTTGTACAGACGCACG

### 2.7 Statistical analysis

Categorical variables were reported as number (%), while continuous variables were reported as mean ± standard deviation (SD) or median (interquartile range, IQR), if the distribution was normal or non-normal, respectively. Continuous variables between groups were compared using the one-way analysis of variance (ANOVA) followed by a *post-hoc* pairwise Tukey’s honestly significant difference (HSD) or Kruskal-Wallis tests, as appropriate. Paired samples were compared with a paired t-test or the nonparametric Wilcoxon signed-rank test when appropriate. Chi-squared test was performed for categorical comparisons.

Receiver operating characteristic (ROC) curves (*) were constructed to determine the best cut-point for BPI to predict the PA phenotype or resistance to ciprofloxacin. Youden’s index (Youden, 1950) was defined for all points along the ROC curve, and the maximum value of the index was used as a criterion for selecting the optimum cut-off point. To determine the predictive capacity of BPI for identification of PA mucoid phenotype or resistance to ciprofloxacin, we determined sensitivity, specificity, positive and negative predictive values (*), along with the 95% confidence intervals (CIs).

All statistical analyses were performed with the SPSS program version 22.0. The level of significance was adjusted at 0.05 (two-tailed).

## 3 Results

### 3.1 Patients and strains

Sixty-nine BE patients were included (25 of them with BE-COPD). Forty-eight patients had been chronically infected by PA for a period of 5.5 [2.25-12] years whilst 21 were intermittently colonized. Thirty-three mucoid and 36 non-mucoid PA isolates similarly distributed between the BE-COPD and BE (p=0.78) were found. The distribution of mucoid vs non-mucoid PA strains was different between chronically infected and intermittently colonized patients (94% vs 6% for the mucoid strains and 47.2% vs 52.8% for the non-mucoid strains, respectively, p < 0.001).

### 3.2 Gram visualization

**Figure 1:**
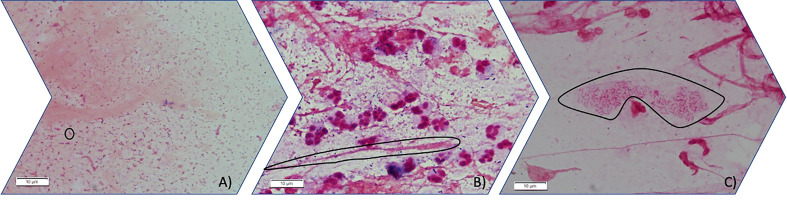
Three different stages of PA mode of growth in sputum observed by light microscopy (100X). **(A)** Planktonic stage in which Gram-negative bacilli are seen free floating in planktonic mode of growth (circled an individual gram-negative bacilli). **(B)** Gram-negative bacilli grow aggregated forming immature biofilms. (circled an aggregate of gram-negative bacilli inside mucus) **(C)** Gram-negative bacilli can be found aggregated embedded in an optically distinguishable alginate extracellular matrix circled in the image.

Presence of alginate in sputa Gram stains was observed in 44% of the patients with a chronic PA respiratory infection, but was not found in the non-chronically infected patients (p = 0.004). Alginate was more frequently when the mucoid PA was isolated than when the non-mucoid PA was isolated (93.3% vs 6.7%, respectively, p < 0.001) and more frequently observed in the non-MDR PA isolates than in the resistant ones (MDR and XDR) (78.6% vs 21.4%, respectively, p = 0.010). The presence of alginate in the Gram stains did not show a statistically significant association with differences in the BPI, although the sputum samples with alginate were associated with lower BPI values at 5 h than those without alginate (13.38 [6.98-19.73] vs 16.7 [7.18-20.00], p = 0.202) (**Figure 1**).

### 3.3 Biofilm ring test

**Figure 2:**
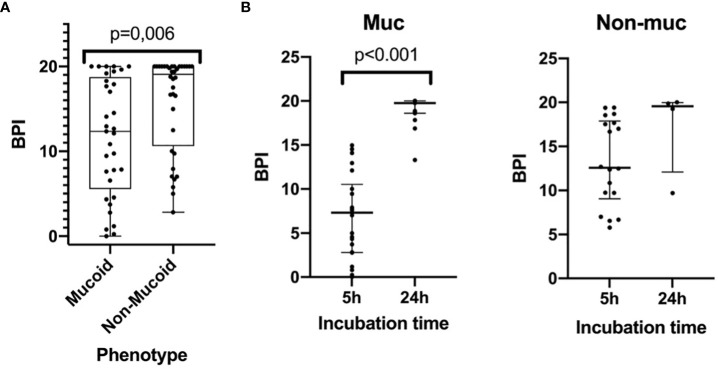
The Biofilm index of mucoid and non-mucoid PA phenotypes and its time-dependent increase during incubation. **(A)** Boxplot showing BPI of the 69 PA isolates, by mucoid and non-mucoid PA, read at 5h as recommended by manufacturer. Median and interquartile ratio are represented by the box and whiskers show the maximum and minimum values. The non-mucoid vs mucoid PA phenotype is associated with an increased BPI (19,08 [10,63-20,00] vs 12,36 [5,55-18,74] p=0.006, respectively). **(B)** BPI at 5 vs 24h by phenotype. Median is represented by the central line whilst interquartile ratio is represented by the two lines at the extremes. A statistically significant increase of the BPI can be seen in mucoid strains when incubating at 24 h whilst a greater heterogenous non-statistically significant result is achieved in non-mucoid strains).

The BPI after 5 h was significantly different between the mucoid and non-mucoid strains, being higher in the non-mucoid isolates (12.36 [5.55-18.74] vs 19.08 [10.63-20.00], respectively, p = 0.006) (**Figure 2A**). In a subset of 22 mucoid and 18 non-mucoid PA strains with slow growth, BPI was compared at 5 h vs 24 h of incubation. The percentage of BPI achieved at 5 h was different between mucoid and non-mucoid strains (35.33 ± 23.78 vs. 68.37 ± 23.07 ± 23.07, p<0.0001), which demonstrated that the delay in growth was superior in mucoid than in non-mucoid strains. The BPI increased in a time-dependent manner for mucoids at 5 vs 24h (4.55 [1.18-7.86] vs 19.75 [18.60-20.00], respectively, p = 0.001). By contrast, no statistically significant increase in the BPI was found for non-mucoid strains at 5 h vs 24 h (6.84 [6.01-9.05] vs 19.55 [12.09-19.96], respectively, p = 0.068) (**Figure 2B**).

**Figure 3:**
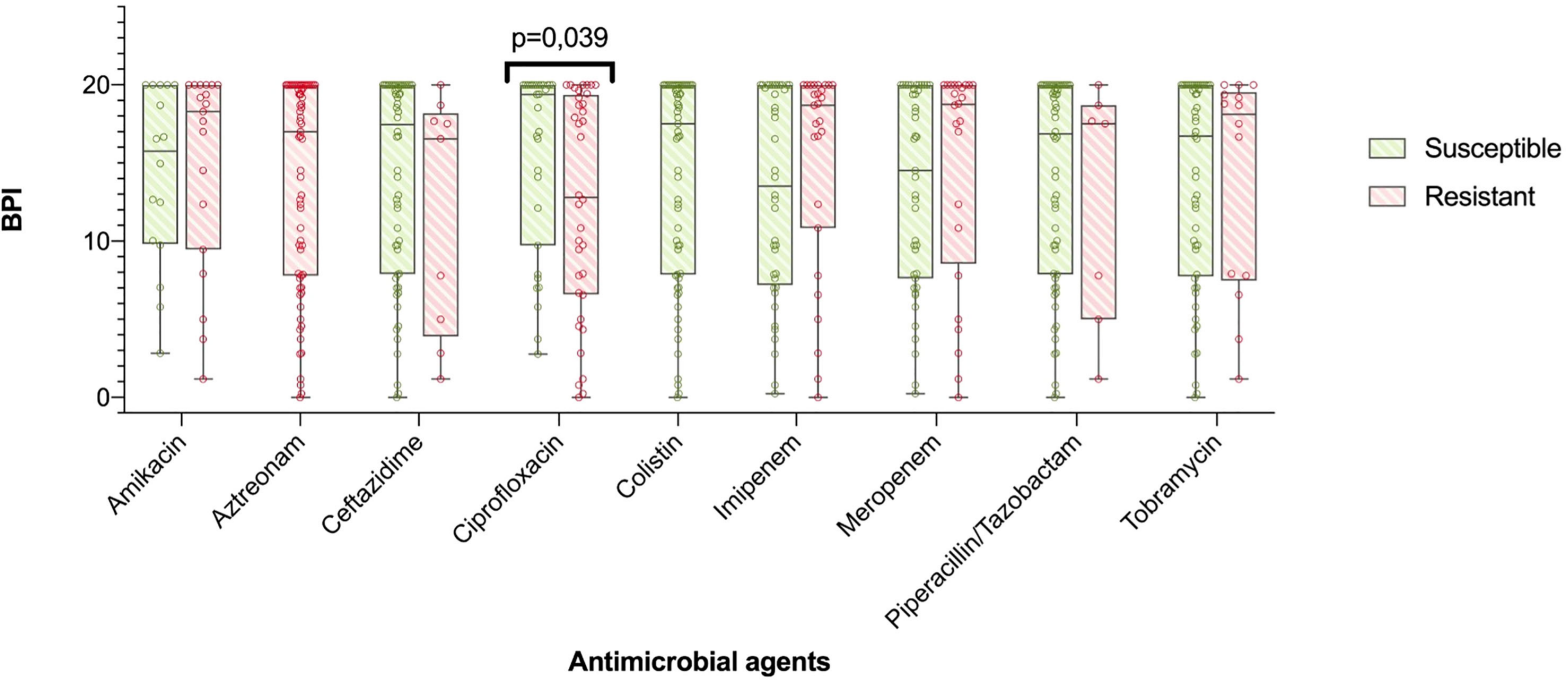
BPI in accordance to antibiotic resistance. The boxplot represents median and interquartile ratio, whiskers represent minimum and maximum values. Differences in the BPI in accordance to antibiotic resistance pattern where only statistically significant comparing ciprofloxacin resistant vs. susceptible PA (12,94 [6,58-19,37] vs. 19,38 [9,73-20,00], p=0.039, respectively).

In particular, all the 8 mucoid strains with a low BPI that were weak biofilm producers at 5 h exhibited a strong and very strong biofilm producer phenotype at 24 h based on their BPI (1.98 [0.38-4.19] vs 19.31 [17.13-20.00], respectively, p = 0.012). Additionally, the categorical stratification of the BPI (at 5 h) into weak, moderate, strong and very strong biofilm producers presented significant differences when comparing mucoid and non-mucoid PA strains (p = 0.022). After 24 hours, 85% of the mucoid vs 56% of the non-mucoid strains demonstrated a strong or very strong ability to produce biofilms (see **Table 2**). Comparing the BPI of resistant and susceptible strains for all the antimicrobial agents, no significant differences were found in the BPI, except for ciprofloxacin. Ciprofloxacin-resistant strains presented a lower BPI than susceptible strains (12.94 [6.58-19.37] vs 19.38 [9.73-20.00], respectively, p = 0.039) (**Figure 3**).

**Table 2:** Categorical stratification of the BPI among mucoid and non-mucoid *Pseudomonas aeruginosa* strains.

		5h (n=69)		24h (n=29)	
		Phenotype			
		Mucoid	Non-Mucoid	Mucoid	Non-Mucoid
Biofilm production	Weak	8 (24.2%)	2 (5.6%)	0 (0%)	1 (11.1%)
	Moderate	6 (18.2%)	6 (16.2%)	0 (0%)	1 (11.1%)
	Strong	7 (21.2%)	3 (8.3%)	3 (15.0%)	2 (22.2%)
	Very strong	12(36.4%)	25(69.4%)	17 (85.0%)	5 (55.6%)

Although a trend of an increased BPI was found in the PA isolates from intermittently colonized patients compared to those from chronically infected patients, this was not statistically significant (15.27 ± 1.21 vs 13.24 ± 1.00, respectively, p = 0.20). PA isolates from patients with BE and from those with BE-COPD did not differ in their BPI (13.47 ± 1.076 vs 14.55 ± 1.09, respectively, p = 0.49). No differences were found in the categorical stratification of the BPI when comparing the chronicity of infection (intermittent vs chronic PA infection), resistance pattern (MDR or XDR vs non-MDR) or the underlying respiratory disease (BE or BE-COPD).

### 3.4 MucA mutations

**Figure 4:**
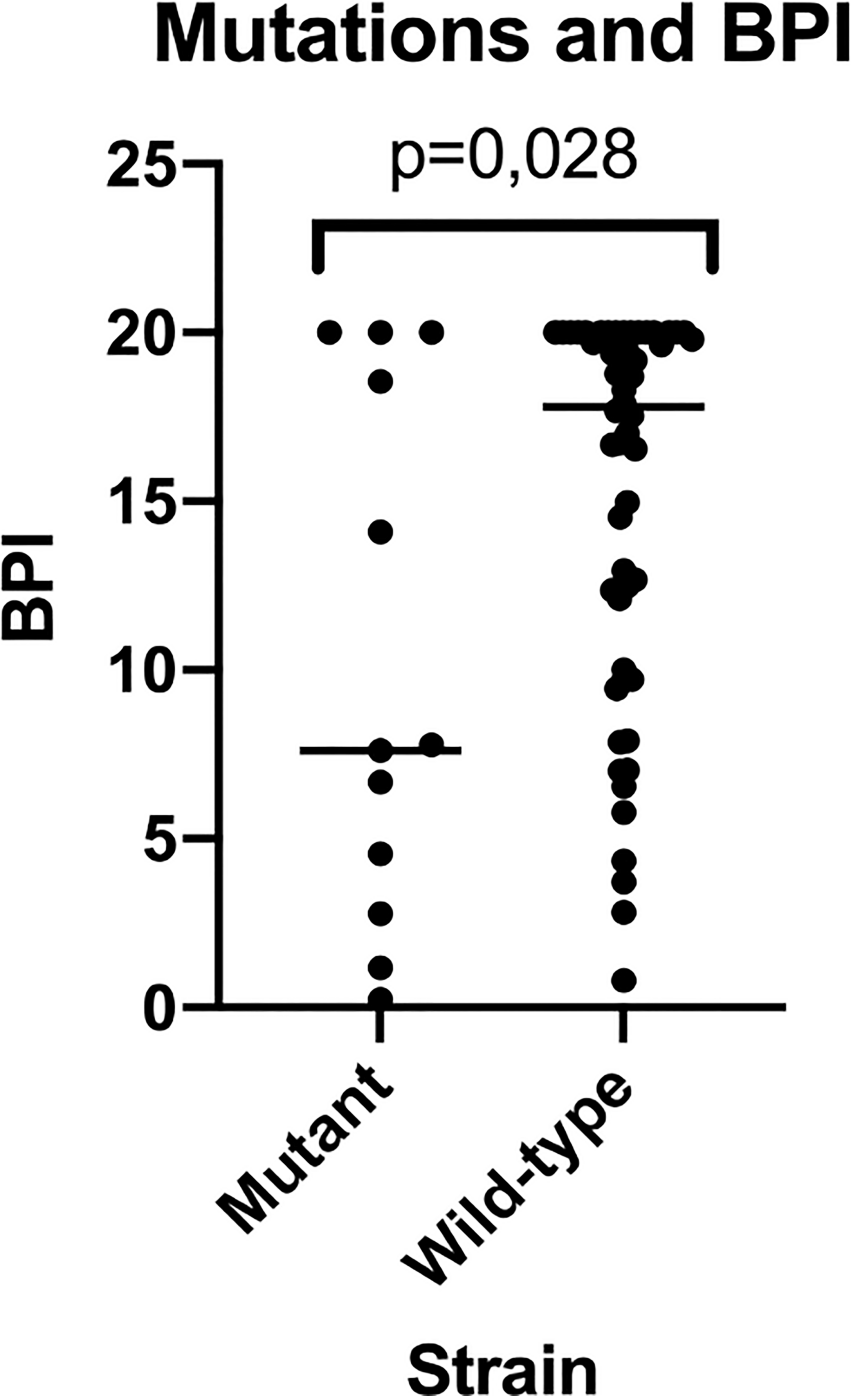
BPI of mutant vs wild-type strains for mucA gene. This figure shows how mutant strains present a reduced BPI when compared to wild-type strains being this last population much more heterogeneous in BPI testing results.

Out of the 67 PA strains tested, 13 had mutations in the mucA gene (mutant mucA), whilst 54 did not (wild-type mucA). The BPI at 5 h of the mutants was lower compared to the wild-type PA strains (7.61 [1.98-19.27] vs 17.79 [9.94-20], respectively, p = 0.028) (**Figure 4**). In contrast, at 24 h, differences were not statistically significant between the mutants vs. the wild type BPI (20.00 [18.80-20.00] vs 18.64 [15.79-19.59], respectively, p = 0.051).

Interestingly, 50% of the strains that showed slow growth (BPI differed at 5 h vs 24 h) presented mutations in the mucA gene, whilst all the non-slow-growing strains had the wild-type mucA gene (p = 0.005). Wild-type strains presented an increased proportion of resistance compared to the mucA mutant strains (87.9% vs 12.1%, respectively, p = 0.05). In particular, amikacin resistance was higher in the wild-type strains than in the mutant PA strains (84.2% vs 15.8%, p = 0.032).

No differences were found in the distribution of the mucoids vs non-mucoids in mutant and wild-type PA strains, BE vs BE-COPD, intermittent vs chronic PA colonization or the 3 categories of Gram.

### 3.5 Ciprofloxacin resistance mechanism

Twenty four out of 43 (56%) PA isolates did not present any mutation on QRDR genes and they were quinolone susceptible as confirmed by disc diffusion (group 0). Five out of 43 (12%) presented <3 mutations on QRDR genes (group 1) and they were quinolone resistant as confirmed by disc diffusion with and an average MIC of 9 mg/L. Finally, 14 out of 43 (33%) presented ≥3 mutations on QRDR genes (group 2) and they were quinolone resistant as confirmed by disc diffusion and with an average MIC of 20-32mg/L. Comparing those strains of group 0, group 1 and group 2 we observed that the group 2 ones had the lowest BPI (17.64 [7.68-2.00], 12.67 [10.91-20.00] and 8.20 [3.45-13.88], p=0.030, respectively). Pairwise comparisons found significantly lower BPI in group 2 compared with group 0 (p=0.012) without any other statistically significant differences.

### 3.6 Predictive performance of BPI compared to phenotype or resistance to ciprofloxacin

**Figure 5:**
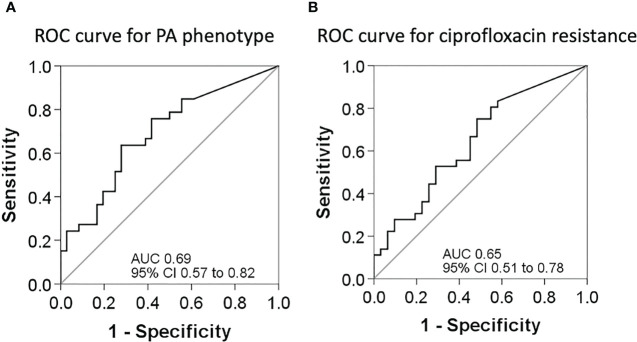
ROC curves for sensitivity and specificity. **(A)** ROC curve to assess the best cut-off point of the BPI for PA phenotype determination. **(B)** ROC curve to assess the best cut-off point of the BPI for PA resistance to ciprofloxacin.

Following Youden’s index methodology, we selected 14.75 as the optimal cut-off point for BPI to predict the PA phenotype (<14.75 = Mucoid, ≥14.75 = Non-mucoid; sensitivity, specificity, positive and negative predictive values were 64% [95% CI 46% to 82%], 72% [95% CI 56% to 88%], 68% [95% CI 50% to 86%], and 68% [95% CI 52% to 85%], respectively), and 19.28 as the optimal cut-off point for BPI in relation to resistance to ciprofloxacin (<19.28 = Ciprofloxacin resistant, ≥19.28 = Ciprofloxacin susceptible; sensitivity, specificity, positive and negative predictive values were 75% [95% CI 59% to 91%], 52% [95% CI 32% to 71%], 64% [95% CI 49% to 80%], and 64% [95% CI 43% to 85%], respectively) (**Figure 5**).

## 4 Discussion

To the best of our knowledge, this is the first study that validates BioFilm Ring Test^®^ (BRT) in a significant number of PA strains from patients with bronchiectasis and which describes BioFilm production Index (BPI) associations with microbiology and clinical outcomes. We found that at 5h of incubation, non-mucoid PA and ciprofloxacin susceptible showed higher BPI than mucoid and ciprofloxacin resistant PA strains, respectively; We suggested a new application of BRT using BPI cut off points to predict the mucoid (<14.75) or Ciprofloxacin resistant (<19.28) phenotype, two outcomes of clinical interest in the context of chronic respiratory diseases. In addition, the presence of alginate in Gram images was associated with the mucoid phenotype, non-MDR PA strains and with the chronic respiratory infection status of the patient. Considering the current lack of routine methods in hospital settings to diagnose biofilm and their clinical implications, this translational approach reveals new diagnostic applications for BRT.

**Figure 6:**
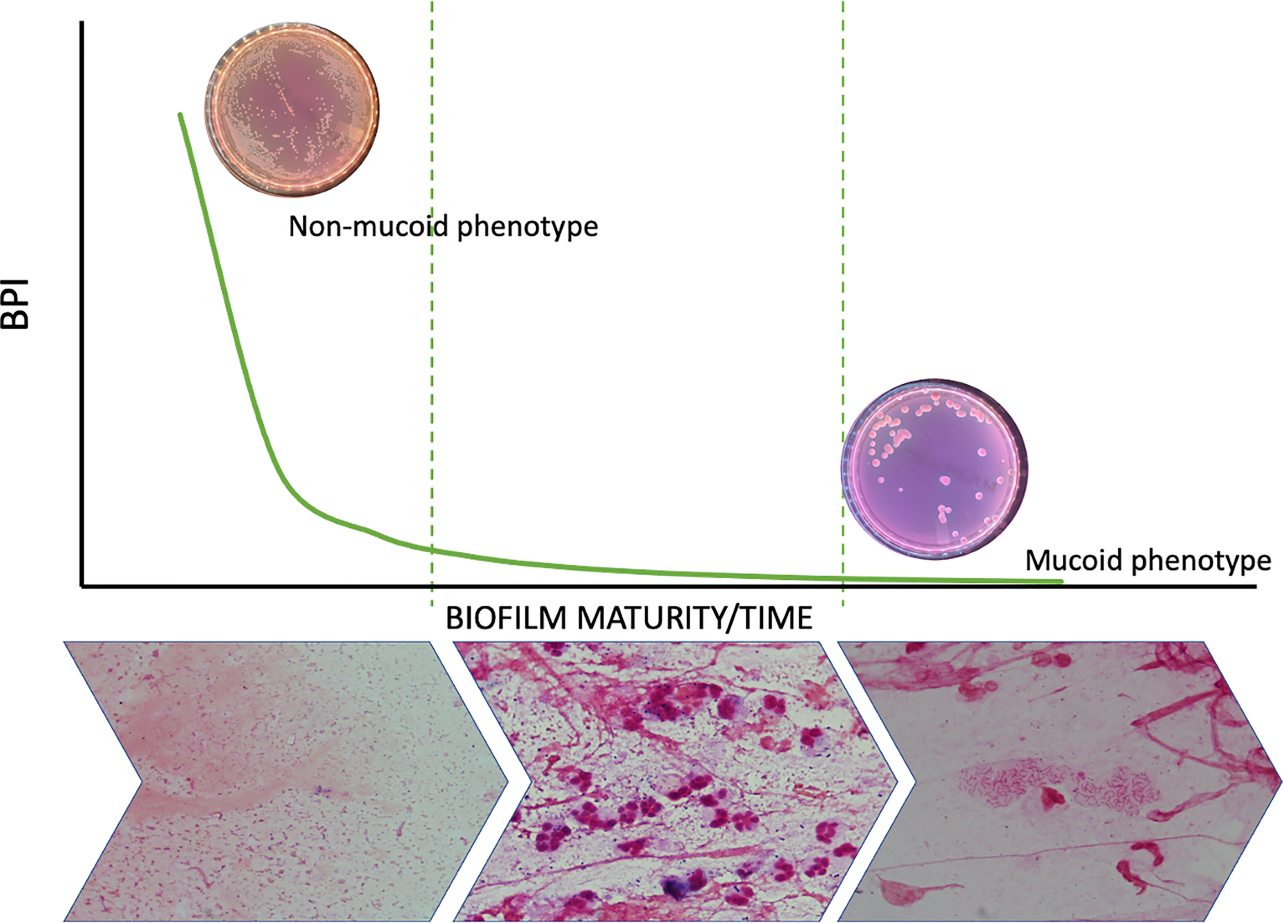
Biofilm dynamic during time and phenotype switch. The figure shows how the metabolic ratio of PA decreases whilst phenotype switches from a non-mucoid state to a mucoid phenotype and at the same time PA starts growing in biofilms instead of being found in planktonic state.

Biofilm formation is a dynamic process that occurs in the first phase, when strains switch from the planktonic to a sessile mode of growth in which they increase their production of adhesins (Youden, 1950), grow in aggregates and are regulated by quorum-sensing signaling pathways (Berne et al., 2015). Biofilm maturation in human PA infections involves the overproduction of extracellular matrix, which is carried out by mucoid strains, and a sustained lethargic metabolism (**Figure 6**). We found that mucoid PA strains, associated with mature biofilms, presented a lower BPI compared to non-mucoid strains at 5 h. However, when the BRT was extended to 24 h, all the mucoid strains exhibited the highest BPI score, indicating that the performance of the mucoid PA strains in the BRT was influenced by the downregulated metabolism of the mucoid phenotype, which needed extra time to reveal their true BPI. Further analyses demonstrated that a BPI < 14.75 predicted the mucoid phenotype with 64% sensitivity and 72% specificity at 5 h. Such values could be, hopefully, improved with a greater sample size.

Interestingly, despite no differences were found for other antibiotics, ciprofloxacin resistant PA presented lower BPI than susceptible strains as it has already been seen in E. coli for extensive spectrum beta lactamase (Mukherjee and Bassler, 2019). This is important since ciprofloxacin is the first recommended treatment for *Pseudomonas aeruginosa* eradication in bronchiectasis patients (Polverino et al., 2017). In spite of this, the mucoid PA has been previously linked to a more susceptible antimicrobial profile compared to the non-mucoid (Batoni et al., 2019). Here we found that PA isolates with more than 3 mutations on QRDR genes were those exhibiting the lowest BPI. Based on our data, the observed correlation between decreased BPI and ciprofloxacin resistance can be explained by an increase in the number of mutations in QRDR and the fitness-cost associated to these mutations. Strains exposed to high levels of environmental stress are more likely to undergo mutations in QRDR, particularly when their metabolism is downregulated. This downregulation could account for the observed link between quinolone resistance and decreased BPI at the 5-hour time point. Further investigation into the use of BRT to predict ciprofloxacin resistance is warranted, given that the traditional method requires a turnaround time of at least 48 hours, whereas BRT can provide results in as little as 5 hours. Recent research has shown that subinhibitory concentrations of β-lactams can induce the BioFilm index (Fernández-Barat et al., 2017), highlighting the potential of BRT as a promising diagnostic tool for Pseudomonas respiratory infections

Herein, we describe for the first time the association between mutations in mucA gene and BPI performance. Interestingly, our results are in accordance to what was observed for the mucoid phenotype. As mucoids, the 13 mutant PA presented a time dependent increase in BPI at 5 h to 24h. In contrast, wild type PA, as well as non-mucoid, presented a less time dependent BPI. In addition, we confirmed slow growth was associated with mucA mutations.

The lack of differences found in the BPI of patients with BE-COPD and BE is attributed to the fact that mucoid and non-mucoid were similarly distributed between BE-COPD and BE alone. Our findings are in line with previous reports indicating that the presence of BE does not influence mortality in long-term follow-up hospitalized COPD exacerbations (Olivares et al., 2020). Thus, the underlying respiratory disease may not have such a relevant role on PA phenotype and biofilm production which rather responds to the stage of chronic infection.

A limitation of this study was the variability in metabolic rates between PA strains, with some strains growing faster than others. To overcome this limitation, we assessed BRT at two different time points. It is also important to note that while we tested the BRT for PA, as it is the most common pathogen in our population, this test could also be applied to other biofilm producing microorganisms.

## 5 Conclusions

The BioFilm Ring Test^®^ (BRT) is a promising technology that can be integrated into clinical practice due to its ability to rapidly assess the biofilm-forming capabilities of microorganisms within just 5 hours. While further validation is needed to assess its predictive value for the mucoid phenotype and the ciprofloxacin resistance, the BRT has the potential to shed light on biofilms that are currently underestimated in antimicrobial stewardship efforts.

## Data availability statement

The original contributions presented in the study are included in the article/**Supplementary Material**. Further inquiries can be directed to the corresponding authors.

## Ethics statement

The studies involving human participants were reviewed and approved by the Internal Review Board of the Hospital Clinic of Barcelona (registry number HCB/0236). Written informed consent was obtained from all patients.

## Author contributions

LF-B and NV designed and performed the experimental work, analyzed the results and wrote the manuscript. AT, RA, AP, AC, VA, LB-F and PO contributed to the clinical follow-up and sample collection of BE patients. RL-A and RC did the genetic analysis on mucA. AM and AG assisted on the statistics and figure design. LM and JV assisted on the PA confirmation by MALDI and CP and TB assisted in the BPI technology and biofilm data collection. All authors reviewed the present manuscript before publication.
